# Variation of Nucleophilicity *N*_H*n*A_ of Atoms A in Hydrides H_*n*_A with the Group and Row of A in the Periodic Table

**DOI:** 10.1002/cphc.202500030

**Published:** 2025-04-28

**Authors:** Ibon Alkorta, Anthony Legon

**Affiliations:** ^1^ Instituto de Química Médica (IQM‐CSIC) Juan de la Cierva, 3 E‐28006 Madrid Spain; ^2^ School of Chemistry University of Bristol Cantock's Close Bristol BS8 1TS UK

**Keywords:** dissociation energies of hydrogen‐bonded complexes of Lewis bases H_*n*_A, dependence of nucleophilicity of Group and Row in Periodic Table, nucleophilicities of hydrides of Group 13‐17 atoms in Periodic Table

## Abstract

Nucleophilicities *N*_H*n*A_ of hydrides of atoms A acting as hydrogen‐bond acceptors in complexes H_*n*_A···HX are reported. The H_*n*_A initially chosen are HB, H_2_C, H_3_N, H_2_O, and HF, that is from groups 13, 14, 15, 16, and 17, respectively, of row 1 of the Periodic Table. The Lewis acids HX involved are HF, HCl, HBr, HI, HCCH, and HCP. Nucleophilicities are determined from dissociation energies *D*_e_ for the process H_*n*_A···HX = H_*n*_A + HX by using *D*_e_ = *cN*_H*n*A_.*E*_HX_, where *E*_HX_ are electrophilicities of the Lewis acids HX. This procedure is also followed for hydrides H_*n*_A in which the atoms A are from the same groups, but from rows 2, 3, and 4 of the Periodic Table. The order of *N*_H*n*A_ values found is row 1 > row 2 ≈ row 3 ≈ row 4 in each of groups 13, 15, 15, 16, and 17, with a large decrease from row 1 to row 2 but with small decreases from rows 2 to 3 to 4. The series Rg···HX is similarly investigated to give the order of nucleophilicities Rg = Xe > Kr > Ar. > Ne, which is reversed from that in the hydrides of groups 13 to 17.

## Graphical abstract

Gradients of *D*_e_ versus electrophilicity *E*_HX_ graphs for hydrogen‐bonded complexes H_*n*_A···HX give nucleophilicities of atoms A of Lewis base H_*n*_A. The dependence of nucleophilicity of hydrides H_*n*_A on their position in the periodic table is then obtained by varying the atom A down the rows within a group and across rows from group to group.

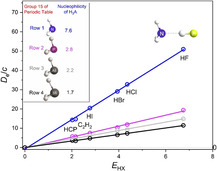

## 1 Introduction

Ingold introduced the word “nucleophilic” (nucleus‐seeking) [and its partner “electrophilic” (electron‐seeking)] into chemistry in 1933.^[^ ^1^ ^]^ Several scales of nucleophilicity have since been proposed. These have been based mainly on the effects of a nucleophilic region of a reagent molecule on the rate of a chemical reaction in which the nucleophilic center attacks an electrophilic region of another molecule to bring about reaction. Early among proposed scales of nucleophilicity is that associated with the names of Swain and Scott^[^ ^2^ ^]^ and the equation proposed by Ritchie.^[^ ^3^ ^]^ In more recent times, Mayr and coworkers^[^ ^4^ ^]^ have carried out important and wide‐ranging research, including an article entitled “Scales of Nucleophilicity and Electrophilicity: A System for Ordering Polar Organic and Organometallic Reactions.” The Mayr group also maintains a website that carries much data of interest concerned with scales of nucleophilicity and electrophilicity.^[^ ^5^ ^]^ An extensive (and regularly upgraded) review^[^ ^6^ ^]^ is a rich source of information about electrophilicity scales.

The purpose of this article is to determine the nucleophilicity of atom A in hydride molecules H_*n*_A as a function of (1) the group of the Periodic Table in which atom A of the Lewis base resides and (2) the row of the Periodic Table in which atom A resides. To this end, we shall use a different definition of nucleophilicity from those based on rates of reaction mentioned earlier. The definition chosen here depends on the propensity of a nucleophile to form a hydrogen‐bonded complex, that is for the nucleophile to interact with an electrophile, namely the H atom of an acid HX. This propensity was first defined^[^ ^7^ ^]^ in terms of one measure of the strength of the complex, namely the intermolecular stretching force constant *k*_*σ*_ of the complex, by means of the expression $k_{\sigma }=c'N_{B}E_{\text{HX}}$, in which *N*_B_ is the nucleophilicity of the Lewis base B, and *E*_HX_ is the electrophilicity of the Lewis acid HX. It was later shown^[^ ^8^ ^]^ that, to a good degree of approximation, *k*_*σ*_ is directly proportional to a more familiar measure of binding strength, the equilibrium dissociation energy *D*_e_ for processes B···HX = B + HX. Given that *D*_e_ values are readily available via ab initio calculations, the equation defining *N*_B_ and *E*_HX_ was recast in terms of *D*_e_ values as

(1) $$D_{e}\;=\;cN_{B}E_{\text{HX}}\;+\;d$$

in which *c* is another constant, conveniently chosen to be 1 kJ mol^−1^ so that when *D*_e_ is calculated in units of kJ mol^−1^, *N*_B_ and *E*_HX_ are dimensionless. The constant *d* has been added to account for the observation that occasionally *D*_e_ is a linear function of the product *N*_B_ *E*_HX_, rather than directly proportional to it.

The aim of the investigation presented here is to obtain the equilibrium dissociation energies *D*_e_ of hydrogen‐bonded complexes H_*n*_A···HX, in which HX is one of the Lewis acids HF, HCl, HBr, HI, HCCH, or HCP and H_*n*_A is a hydride of an atom A belonging variously to groups 13 to 17 and rows 1 to 4 of the Periodic Table, as summarized in **Table** **1**. In each of the hydrogen‐bonded complexes H_*n*_A···HX referred to, atom A is adjacent to the hydrogen atom of HX and therefore directly involved in the formation of the hydrogen bond. Within a given group, the complexes in rows 2 to 4 have similar angular geometries, while those associated with row 1 can be a little different from those in rows 2 to 4.

**Table 1:** Hydrides of groups 13 to 17 and rows 1 to 4 of the Periodic Table.

Row	Group				
	13	14	15	16	17
1	HB	H_2_C	H_3_N	H_2_O	HF
2	HAl	H_2_Si	H_3_P	H_2_S	HCl
3	HGa	H_2_Ge	H_3_As	H_2_Se	HBr
4	HIn	H_2_Sn	H_3_Sb	H_2_Te	HI

*D*_e_ values for the complexes H_*n*_A···HX are to be calculated ab initio. Group 14 hydrides listed are carbenes and have been discussed in a recent publication.^[^ ^9^ ^]^ All of these, except H_2_C, have singlet electronic ground states. The calculations here for complexes H_2_C···HX yield the singlet states, however, and therefore all hydrides of group 14 in Table 1 are in singlet states.

It has been shown in recent publications^[^ ^10^, ^11^, ^12^ ^]^ that it is possible to define the nucleophilicity of Lewis bases (such as the H_*n*_A listed in Table 1) and the electrophilicities *E*_HX_ of the Lewis acids HX, by means of Equation (1). Many *N*_B_ and *E*_HX_ values were initially generated^[^ ^13^ ^]^ by least‐squares fitting of ab initio‐calculated *D*_e_ values to Equation (1), including the set of *E*_HX_ values for HF, HCl, HBr, HI, HCCH, and HCP. Updated, more accurate values for HI and HBr were later reported in ref.[10] The set of *E*_HX_ values employed here were 6.75, 4.35, 3.94, 2.77, 2.16, and 2.02 for X = F, Cl, Br, I, CCH, and CP, respectively. According to Equation (1), a graph of *D*_e_ versus *E*_HX_ for complexes formed from a fixed Lewis base H_*n*_A and each of a series of Lewis acids HX should be a straight line, usually passing through the origin (*d* = 0) but not always and having a gradient *N*_H*n*A_, the nucleophilicity of that Lewis base, as measured by its propensity to form hydrogen bonds. In determining the nucleophilicities *N*_H*n*A_ of the molecules in Table 1, there are two aims. First, we wish to examine how, for those atoms A from a chosen group of the Periodic Table, the nucleophilicity varies with their row in the table. Secondly, we shall investigate how nucleophilicities of hydrides H_*n*_A from a chosen row of the Periodic Table vary with their group.

## 2 Results and Discussion

### 2.1 Variation of the Nucleophilicity *N*_H*n*A_ of Hydrides within a Given Group of the Periodic Table with Row

**Figure 1:**
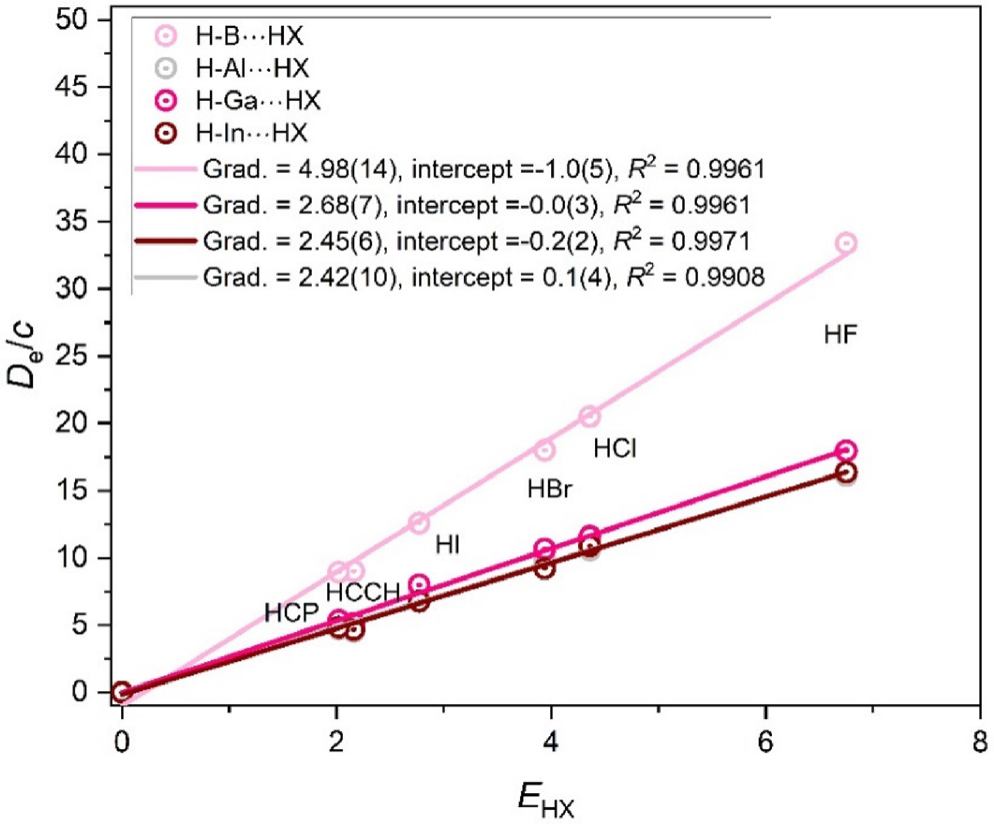
*D*_e_ versus *E*_HX_ for the hydrogen‐bonded complexes H‐A···HX, in which A is one of the group 13 atoms B, Al, Ga, or In (note that *D*_e_ is in units of kJ mol^−1^, and the constant *c* = 1.0 kJ mol^−1^).

Hydrogen‐bonded complexes having group 13 hydrides H‐M (where M = B, Al, Ga, and In) as the proton acceptor have the simple linear arrangement H‐M···HX. The graph of *D*_e_ versus *E*_HX_ for this series of complexes is in **Figure** **1**. Here and in all similar graphs, (0,0) is assumed to be a point, and *c* is set as 1.0 kJ mol^−1^. Because there is evidence from earlier work that HCN behaves anomalously, it is not included in the set (namely HF, HCl, HBr, HI, HCCH, and HCP) of Lewis acids used here.

The gradients of the straight lines in Figure 1 yield the nucleophilicities of the four H‐A Lewis bases, in which A is either a boron, aluminum, gallium, or indium atom. Clearly, the axial nonbonding electron pair carried by the group 13 atom is considerately more nucleophilic for A = boron than in the other three diatomic molecules HA. Indeed, within the errors of the fitted gradients, the *N*_HA_ values for A = Al, Ga, and In are equal but are only half that for A = boron. Thus, within group 13 of the Periodic Table, the first‐row atom B in Lewis bases H‐A has nucleophilicities in the order *N*_H‐B_ > *N*_H‐Al_ ≈ *N*_H‐Ga_ ≈ *N*_H‐In_.

**Figure 2:**
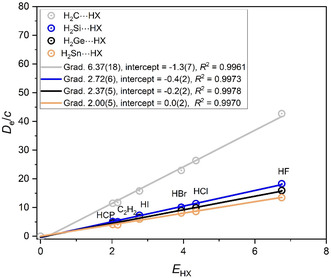
*D*_e_ versus *E*_HX_ for the hydrogen‐bonded complexes H_2_A···HX, in which A is one of the group 14 atoms C, Si, Ge, or Sn (note *D*_e_ is in units of kJ mol^−1^, and the constant *c* = 1.0 kJ mol^−1^).

A similar pattern exists among the carbenes H_2_A (A = C, Si, Ge, Sn) when forming hydrogen bonds with HX molecules, as is evident from the *D*_e_ versus *E*_HX_ graphs displayed in **Figure** **2** (a slightly different version of Figure 2 was published earlier in ref. [9]) Thus, Figure 2 reveals the order of nucleophilicities to be *N*_H2C_ > *N*_H2Si_ ≈ *N*_H2Ge_ ≈ *N*_H2Sn_. Again, the largest nucleophilicity is associated with the row 1 atom of group 14 while those in the remainder of the group are much smaller and nearly equal.

**Figure 3:**
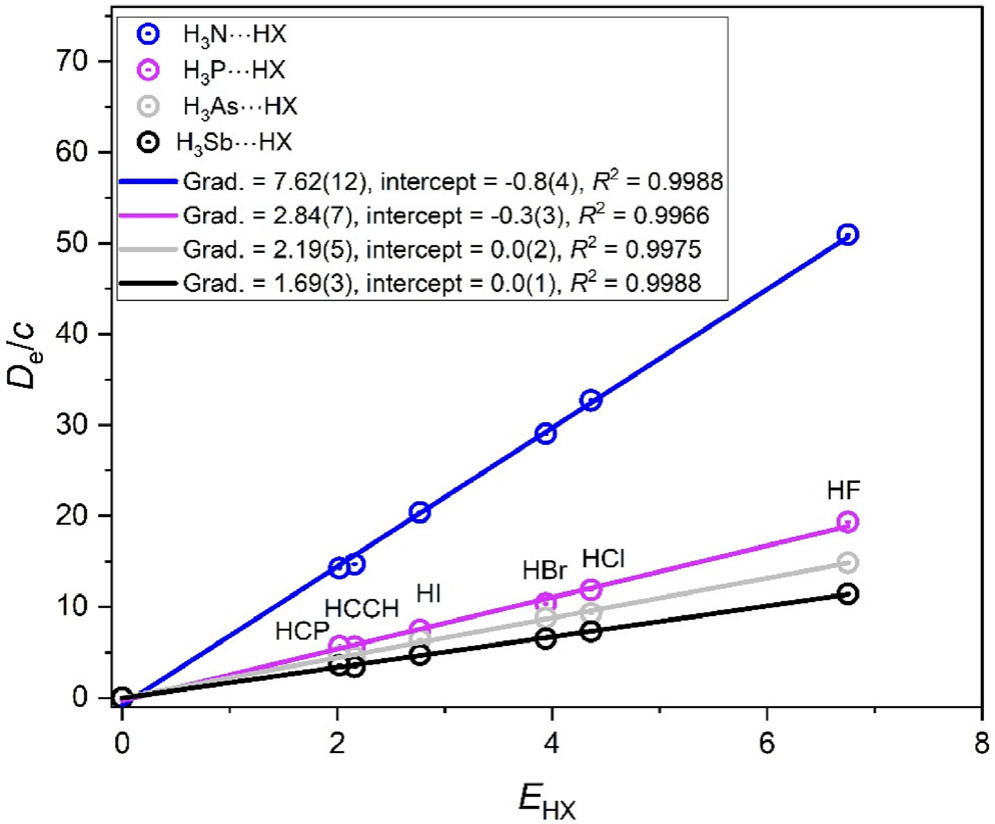
*D*_e_ versus *E*_HX_ for the hydrogen‐bonded complexes H_3_A···HX, in which A is one of the group 15 atoms N, P, As, or Sb (note that *D*_e_ is in units of kJ mol^−1^, and the constant *c* = 1.0 kJ mol^−1^).

The same trend in the nucleophilicity of the axial nonbonding electron pair carried on the *C*_3_ axes of the symmetric‐top Lewis bases H_3_A (in which A is one of the group 15 atoms N, P, As, or Sb) is revealed by examination of the corresponding *D*_e_/*c* versus *E*_HX_ graphs shown in **Figure** **3**. Thus, it is concluded that the order of the nucleophilicities is *N*_H3N_ > *N*_H3P_ ≈ *N*_H3As_ ≈ *N*_H3Sb_. The Lewis base containing the atom from the first row of the Periodic Table is again associated with the greatest nucleophilicity, while the group 15 atoms in rows 2, 3, and 4 have smaller, nearly equal values.

**Figure 4:**
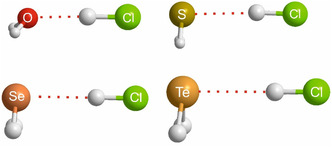
Optimized geometries of the hydrogen‐bonded complexes H_2_A···HCl (A = O, S, Se, Te) calculated at the CCSD(T)(F12c)/cc‐pVDZ‐F12 level of theory (approximately to scale).

**Figure 5:**
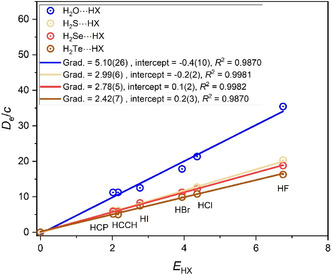
*D*_e_ versus *E*_HX_ for the hydrogen‐bonded complexes H_2_A···HX, in which A is one of the group 16 atoms O, S, Se, or Te (note that *D*_e_ is in units of kJ mol^−1^, and the constant *c* = 1.0 kJ mol^−1^).

When the hydrides H_2_A of group 16 atoms from rows 1 to 4 of the Periodic Table (namely H_2_O, H_2_S, H_2_Se, and H_2_Te) are investigated in a similar way, there is, however, a complicating factor not previously encountered here. All the complexes discussed thus far within a given group are isostructural. When the geometries of the H_2_A···HX optimized at the CCSD(T)(F12c)/cc‐pVDZ‐F12 level for the hydrides H_2_A of the group 16 atoms (as set out in **Figure** **4** for the examples having HX = HCl) are examined, that of H_2_O···HCl^[^ ^14^ ^]^ is less steeply pyramidal at O than for the remainder. H_2_O···HF is slightly more pyramidal^[^ ^15^ ^]^ but still does not have the rigid right‐angled geometries of the H_2_S,^[^ ^16^, ^17^ ^]^ H_2_Se, or H_2_Te complexes that are seen in Figure 4. This difference in the angular geometry of H_2_O···HX species might be the cause of the slightly inferior least‐squares fit to the points for these complexes shown in **Figure** **5**. Notwithstanding these geometric variations, it is clear from the graphs in Figure 5 that the same pattern of nucleophilicities holds for the group 16 hydrides as it does for those of groups 13, 14, and 15, namely *N*_H2O_ > *N*_H2S_ ≈ *N*_H2Se_ ≈ *N*_H2Te_.

**Figure 6:**
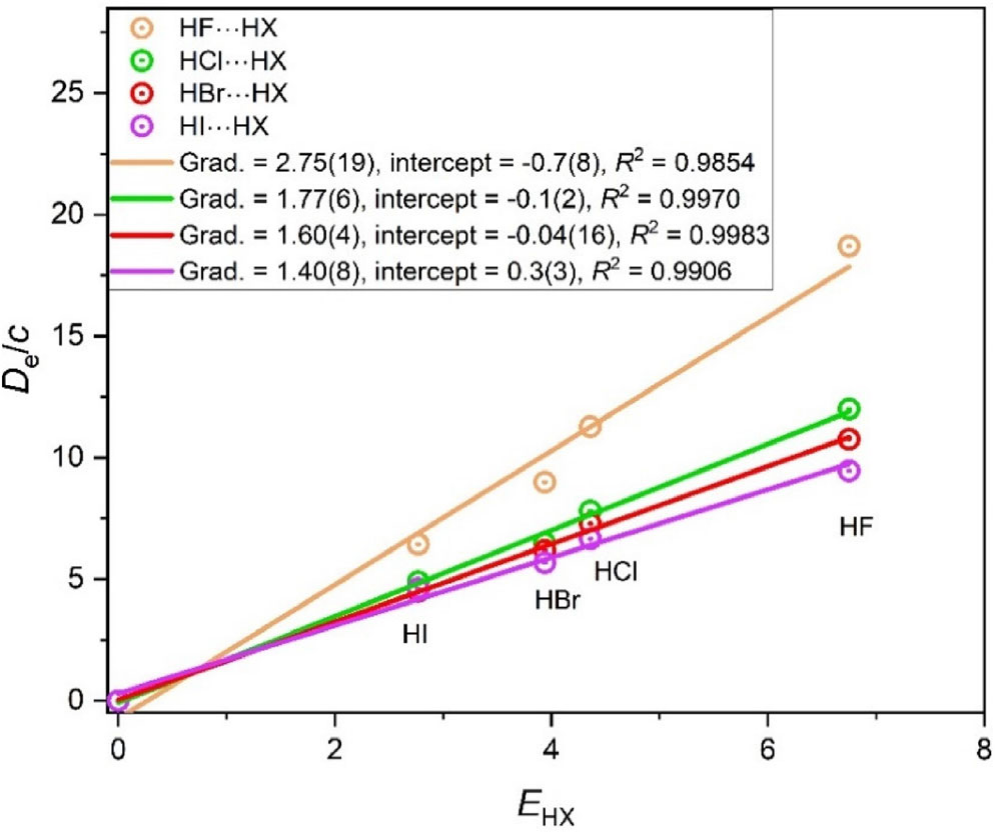
*D*_e_ versus *E*_HX_ for the hydrogen‐bonded complexes HA···HX, in which A is one of the group 17 atoms F, Cl, Br, or I (note that *D*_e_ is in units of kJ mol^−1^, and the constant *c* = 1.0 kJ mol^−1^).

**Figure 7:**
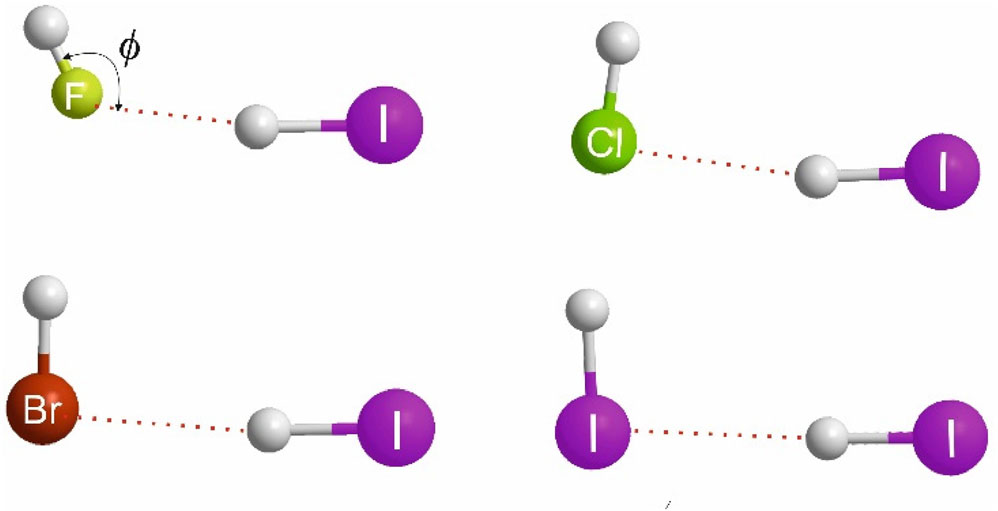
Geometries of HX···HI complexes optimized at the CCSD(T)(F12c)/cc‐pVDZ‐F12 level. The angle *ϕ* is close to tetrahedral for HF···HI, in which F is the proton acceptor, but is closer to a right angle when X ≠ F.

The final set of hydrogen‐bonded complexes involving simple hydrides as the hydrogen‐bond acceptor to be considered here are those HA···HX in which the halogen atoms of rows 1 to 4 of the Periodic Table (i.e., the group 17 atoms A = F, Cl, Br, and I) are the proton acceptors. The graphs of *D*_e_ versus *E*_HX_ for these series of complexes in which the proton donors are HF, HCl, HBr, HI, HCCH, and HCP are in **Figure** **6**. There is, however, a similar problem here to that encountered for the H_2_M···HX (group 16) series. This can be illustrated by the optimized geometries of the various HA···HI complexes, as set out in **Figure** **7**. Thus, while the angular geometry for the HF···HI complex has the angle *ϕ* (as defined for HF···HI in Figure 7) approximately equal to the tetrahedral angle (≈109°), the other members of the series HY···HI have geometries with *ϕ* close to a right angle. Nevertheless, the same pattern in the order of the nucleophilicities, i.e., *N*_HF_ > *N*_HCl_ ≈ *N*_HBr_ ≈ *N*_HI_, is observed in Figure 6 as previously identified in Figure 1, 2, 3, and 5.

### 2.2 Does the Variation of the Nucleophilicities of the Group 18 Atoms (Ne, Ar, Kr, and Xe) with Row in the Periodic Table Follow that of the Hydrides of Groups 13, 14, 15, 16, and 17?

**Figure 8:**
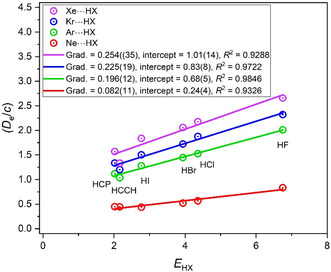
*D*_e_ versus *E*_HX_ for the hydrogen‐bonded complexes A···HX, in which A is one of the group 18 inert gas atoms Ne, Ar, Kr, or Xe (note that *D*_e_ is in units of kJ mol^−1^, and the constant *c* = 1.0 kJ mol^−1^).

**Figure 9:**
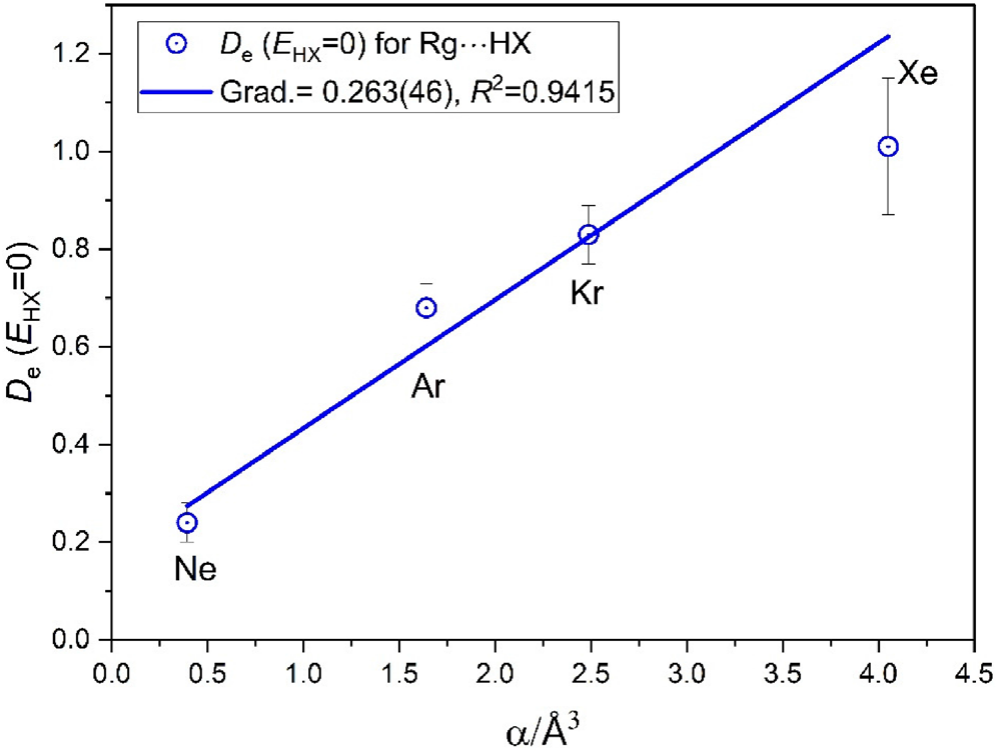
Values of the residual nucleophilicities of rare gas atoms Ne, Ar, Kr, and Xe plotted as a function of the static dipole polarizabilities of the atoms. The residual nucleophilicities are the values of the positive intercepts at *D*_e_(*E*_HX_ = 0) of the *D*_e_ versus *E*_HX_ graphs shown in Figure 8, which is where the electrophilicity of the Lewis acid is hypothetically zero.

**Figure** **8** contains the *D*_e_/*c* versus *E*_HX_ graphs for the complexes Rg···HX, where Rg is Ne, Ar, Kr, or Xe and X = F, Cl, Br, and I. It is immediately clear that the *D*_e_ values are very small compared with those in Figure 1, 2, 3, 5, and 6, with that for Xe···HF being the largest. The order of gradients in Figure 8 is also reversed relative to those established in Section 2.1 for the hydrides of atoms in groups 13 to 17, which presumably have a significant and predominant electrostatic component. Moreover, the intercept at *E*_HX_ = 0 of each line in Figure 8 is a large fraction of the *D*_e_ values and is largest for the Xe···HX series. This implies that the gradients and intercepts might be related (perhaps in a complicated manner) to the static dipole polarizability, α, of the inert gas atom through dispersion and induction interactions. Certainly, α increases for atoms in group 18 with their row in the Periodic Table, with values^[^ ^18^ ^]^ 0.394340(1), 1.6423(1), 2. 487(3), and 4.05(3) Å^3^ for Ne, Ar, Kr, and Xe, respectively. Moreover, **Figure** **9** shows that there is an approximately linear increase in the value of the intercept at *E*_HX_ = 0 as the inert gas changes from Ne, though Ar and Kr, to Xe. This intercept is the residual value of the dissociation energy of the Rg···HX complexes in the hypothetical case in which the electrophilicity of the Lewis acid is zero.

### 2.3 Recasting the Data to Illustrate Graphically the Variation of *D*_e_ with Nucleophilicity *N*_H*n*A_ of Atom A in Hydrides

**Figure 10:**
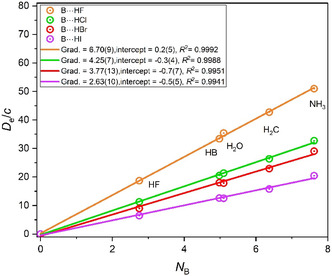
Graphs of *D*_e_/*c* versus *N*_B_ for the molecules B···HX in which B = NH_3_, H_2_C, H_2_O, HB, and HF acting as Lewis bases. HX is fixed in each series, as indicated by color.

It is possible to use the nucleophilicities *N*_H*n*A_ determined in Figure 1, 2, 3, 5, and 6 to show how the values of *D*_e_ vary with *N*_H*n*A_. This requires a graph of *D*_e_ for complexes H_*n*_A···HX plotted against *N*_H*n*A_ in which the Lewis acid HX is fixed while the Lewis base H_*n*_A is varied. No new data is involved. Such graphs for the series of first‐row Lewis bases HB, H_2_C, H_3_N, H_2_O, and HF are in **Figure** **10** for the four cases in which the Lewis acid is fixed as either HF, HCl, HBr, or HI. The gradient of each graph then yields the electrophilicity *E*_HX_ of the Lewis acid concerned. The values are 6.70(9), 4.25(7), 3.7791(3), 2.63(10) for HF, HCl, HBr, and HI, respectively. These agree within two standard errors with the accepted values of 6.75, 4.35, 3.94, and 2.77 used initially in generating the graphs in Figure 1, 2, 3, 5, and 6, as expected.

Figure 10 is also a convenient way to show graphically that the order of the nucleophilicity of the hydrides across the first row is NH_3_ > H_2_C > H_2_O ≈ HB >> HF.

**Figure 11:**
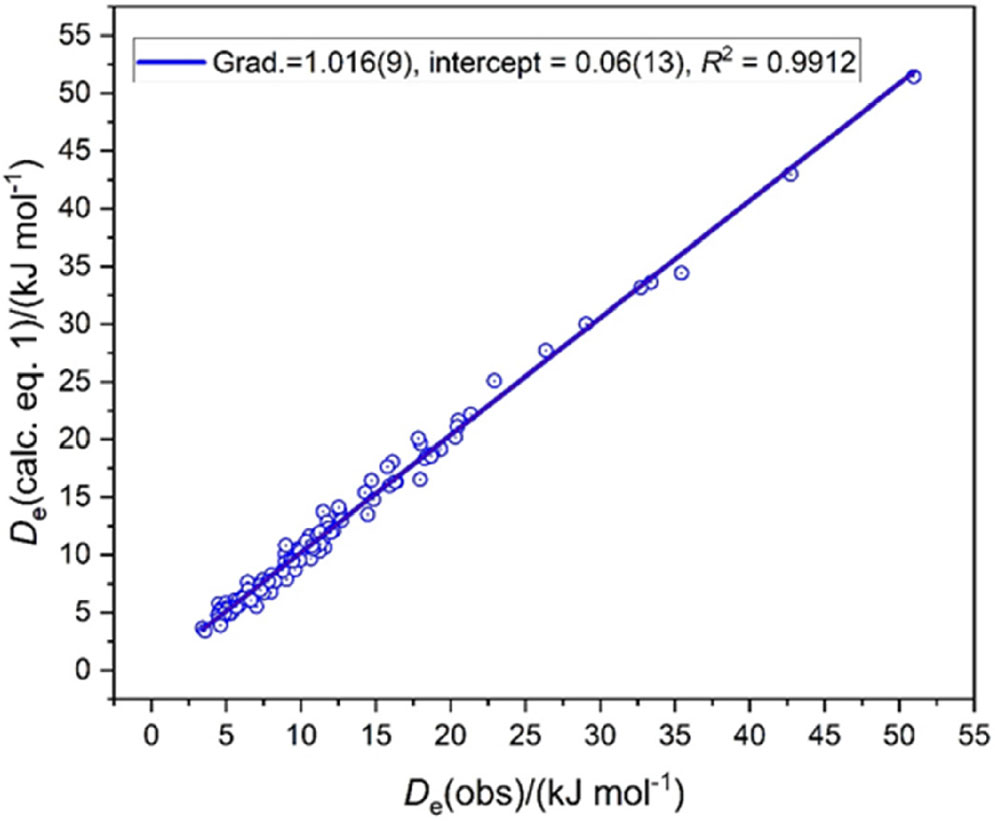
*D*_e_ values calculated at the CCSD(T)‐F12c/cc‐pVDZ‐F12 level versus predictions from *D*_e_ = *cN*_H*n*A_ *E*_HX_ with the *E*_HX_ values and the *N*_H*n*A_ values of the 112 complexes H_n_A···HX discussed in Figure 1, 2, 3, 5, and 6.

From the *E*_HX_ values used for the electrophilicities and the *N*_H*n*A_ values generated in Figure 1, 2, 3, 5, 6, the *D*_e_ values of the 112 H_*n*_A···HX complexes investigated can be predicted with high accuracy using Equation (1), assuming *d* = 0.0, as is clear from **Figure** **11**.

## 3 Conclusions

The hydrides of groups 13, 14, 15, 16, and 17 of the Periodic Table whose nucleophilicities have been investigated in this work are characterized by nonbonding electron pairs that lie along a symmetry axis of the hydride in the case of groups 13, 14, and 15, for which the symmetry axes carry the labels *C*∞, *C*_2_, and *C*_3_, respectively. In each of these cases, the HX molecule lies along a symmetry axis when forming the complex, and therefore it is the nucleophilicity of the nonbonding pair that is important. For the group 16 hydrides, it is known that in H_2_O the two nonbonding pairs and the two O—H bonding pairs are tetrahedrally disposed about O (see Figure 4), while in H_2_S, H_2_Se, and H_2_Te, the bonding pairs and the nonbonding pairs have a right‐angled arrangement, as indicated by the equilibrium geometries shown in Figure 4. For HCl, HBr, and HI, the equilibrium geometries shown in Figure 7 indicate essentially right‐angled geometries, while that involving F of HF as the proton acceptor suggests a tetrahedral disposition. Rules, advanced many years ago, to rationalize angular geometries of hydrogen‐bonded complexes state that in the equilibrium geometry, simple proton‐donor molecules like those considered here lie along the axis of a nonbonding electron pair, as conventionally envisaged.^[^ ^19^, ^20^ ^]^ The angular geometries determined in this study are consistent with these rules. Accordingly, we are led to the conclusion that in our investigations of the group 13, 14, 15, 16, and 17 hydrides, the nucleophilicities that are observed are associated mainly with the nonbonding electron pairs carried by the atom belonging to the group.

Accepting the conclusion of the preceding paragraph, the variation of such nucleophilicities within each group as the row number in the Periodic Table increases from 1 to 4 leads to the interesting result that the nucleophilicity of the hydride (and presumably of the nonbonding pair(s) that is(are) carried by the atom A) is much larger in row 1 and decreases rapidly in row 2 (by a factor of about 2), but then there is little change from row 2 to 3 to 4. The result with the inert gases that constitute group 18 of the Periodic Table is completely different. The order of the nucleophilicities of these spherical, nonpolar atoms is then as follows: row 4 > row 3 > row 2 > row 1, which is just the order of the static dipole polarizabilities of the atoms.

## 4 Experimental Section

The geometries of the systems were optimized at the CCSD(T)‐F12c computational level^[^ ^21^, ^22^ ^]^ with the cc‐pVDZ‐F12 basis.^[^ ^23^ ^]^ Optimized energies and Cartesian coordinates of all complexes investigated are available as Supplementary Material. The CCSD(T) level of the calculation was known as the gold standard for the calculation of medium‐sized systems. The standard frozen‐core approximation was used for all the complexes, except for those containing tin, where 10 electrons were correlated instead of the default (4 electrons). This avoided the mixing of the core and valence electrons in complexes where a tin atom was present. The inherent basis set superposition error was corrected with the Boys and Bernardi full counterpoise method.^[^ ^24^ ^]^ These calculations were carried out with the scientific MOLPRO program.^[^ ^25^ ^]^

## Conflict of Interest

The authors declare no conflict of interest.

## Supporting information

Supplementary Material

## Data Availability

The data that support the findings of this study are available in the supplementary material of this article.
