# Supplementary material for: Variation of Nucleophilicity N HnA of Atoms A in Hydrides H n A with the Group and Row of A in the Periodic Table
Source: Chemphyschem. 2025 Apr 28;26(13):e202500030. doi: 10.1002/cphc.202500030 (PMC12225747; doi:10.1002/cphc.202500030)
Supplement: Supplementary file 1 — Supplementary Material [file CPHC-26-e202500030-s001.pdf]

SUPPLEMENTARY MATERIAL

Variation of Nucleophilicity N_{H_nA} of Hydrides H_nA with the Group and Row of Atoms A within the Periodic Table.

Ibon Alkorta[a] and Anthony C. Legon[b]
[a]Instituto de Química Médica (IQM-CSIC), Juan de la Cierva, 3, E-28006 Madrid, Spain,
[b] School of Chemistry, University of Bristol, Cantock's Close, Bristol BS8 1TS, U. K

Index

Pg. S2-S5 Table S1. Optimised geometries (Å) and energies (au) of HA···HX complexes (A = B, Al, Ga, In) calculated at the CCSD(T)(F12c)/cc-pVDZ-F12 level.

Pg. S6-S9 Table S2. Optimised geometries (Å) and energies (au) of H_2A···HX complexes (A = C, Si, Ge, Sn) calculated at the CCSD(T)(F12c)/cc-pVDZ-F12 level.

Pg. S10-S14 Table S3. Optimised geometries (Å) and energies (au) of H_3A···HX complexes (A = N, P, As, Sb) calculated at the CCSD(T)(F12c)/cc-pVDZ-F12 level.

Pg. S15-S19 Table S4. Optimised geometries (Å) and energies (au) of H_2A···HX complexes (A = O, S, Se, Te) calculated at the CCSD(T)(F12c)/cc-pVDZ-F12 level.

Pg. S20-S23 Table S5. Optimised geometries (Å) and energies (au) of HA···HX complexes (A = F, Cl, Br, I) calculated at the CCSD(T)(F12c)/cc-pVDZ-F12 level.

Pg. S24-S27 Table S6. Optimised geometries (Å) and energies (au) of Rg···HX complexes (Rg = Ne, Ar, Kr, Xe) calculated at the CCSD(T)(F12c)/cc-pVDZ-F12 level.

Pg. S28-S29 Table S7. De (kJ mol $^{-1}$) values calculated at CCSD(T)-F12c/cc-VDZ-F12 level.

Table S1. Optimised geometries (Å) and energies (au) of HA···HX complexes (M= B, Al, Ga, In) calculated at the CCSD(T)(F12c)/cc-pVDZ-F12 level.

HB···HX complexes

HB···HF

CCSD(T)-F12C/CC-PVDZ-F12 ENERGY=-125.61996993

F	0.6057032058	0.0000000000	-2.3255135253
H	0.4415996003	0.0000000000	-1.4033795896
B	0.0824534282	0.0000000000	0.6215287566
H	-0.1297562342	0.0000000000	1.8202949576

HB···HCl

CCSD(T)-F12C/CC-PVDZ-F12 ENERGY=-485.59805775

Cl	0.0000000000	0.0000000000	-1.9728969272
H	0.0000000000	0.0000000000	-0.6779899909
B	0.0000000000	0.0000000000	1.5715067465
H	0.0000000000	0.0000000000	2.7936732651

CCSD(T)-F12C/USERDEF ENERGY=-441.55015868

HB···HBr

Br	0.0000000000	0.0000000000	-3.0760381092
H	0.0000000000	0.0000000000	-1.6368848855
B	0.0000000000	0.0000000000	0.6279907637
H	0.0000000000	0.0000000000	1.8512686605

HB···HI

CCSD(T)-F12C/USERDEF ENERGY=-320.67262861

I	0.0000000000	0.0000000000	-3.4002869071
H	0.0000000000	0.0000000000	-1.7710805256
B	0.0000000000	0.0000000000	0.6557469451
H	0.0000000000	0.0000000000	1.8815180968

HB···HCCH

CCSD(T)-F12C/CC-PVDZ-F12 ENERGY=-102.44437073

H	0.0000000000	0.0000000000	-5.3553391268
C	0.0000000000	0.0000000000	-4.2923743037
C	0.0000000000	0.0000000000	-3.0858915319
H	0.0000000000	0.0000000000	-2.0179386208
B	0.0000000000	0.0000000000	0.7260543070
H	0.0000000000	0.0000000000	1.9542754554

HB···HCP

CCSD(T)-F12C/CC-PVDZ-F12 ENERGY=-404.74033797

P	0.0000000000	0.0000000000	-4.5442489655
C	0.0000000000	0.0000000000	-2.9995678981
H	0.0000000000	0.0000000000	-1.9235451863
B	0.0000000000	0.0000000000	0.8217156745
H	0.0000000000	0.0000000000	2.050001114

HAl···HX complexes

HAl···HF

CCSD(T)-F12C/CC-PVDZ-F12 ENERGY=-342.92846389

F	0.0000000000	0.0000000000	-2.1371381834
H	0.0000000000	0.0000000000	-1.2101742217
Al	0.0000000000	0.0000000000	1.4354229627
H	0.0000000000	0.0000000000	3.0677157329

HAl···HCl

CCSD(T)-F12C/CC-PVDZ-F12 ENERGY=-702.90928539

Cl	0.0000000000	0.0000000000	-1.8348849786
H	0.0000000000	0.0000000000	-0.5499111869
Al	0.0000000000	0.0000000000	2.2850031161
H	0.0000000000	0.0000000000	3.9224077337

HAl···HBr

CCSD(T)-F12C/USERDEF ENERGY=-658.86209888

Br	0.0000000000	0.0000000000	-1.1226556996
H	0.0000000000	0.0000000000	0.3064775543
Al	0.0000000000	0.0000000000	3.1348982278
H	0.0000000000	0.0000000000	4.7734866182

HAl···HI

CCSD(T)-F12C/USERDEF ENERGY=-537.98574907

I	0.0000000000	0.0000000000	-0.8423694861
H	0.0000000000	0.0000000000	0.7804374638
Al	0.0000000000	0.0000000000	3.7321225908
H	0.0000000000	0.0000000000	5.3735217189

HAl···HCCH

CCSD(T)-F12C/CC-PVDZ-F12 ENERGY=-319.75776956

H	0.0000000000	0.0000000000	-5.5666894515
C	0.0000000000	0.0000000000	-4.5036672378
C	0.0000000000	0.0000000000	-3.2977575168
H	0.0000000000	0.0000000000	-2.2322776945
Al	0.0000000000	0.0000000000	1.1134873428
H	0.0000000000	0.0000000000	2.7574281502

HAl···HCP

CCSD(T)-F12C/CC-PVDZ-F12 ENERGY=-622.05383094

P	0.0000000000	0.0000000000	-4.6537600796
C	0.0000000000	0.0000000000	-3.1098710233
H	0.0000000000	0.0000000000	-2.0359215824
Al	0.0000000000	0.0000000000	1.2865857883
H	0.0000000000	0.0000000000	2.9304904903

HGa···HX complexes

HGa···HF

CCSD(T)-F12C/USERDEF ENERGY=-359.72834602

H	0.0000000000	0.0000000000	-2.3655050971
Ga	0.0000000000	0.0000000000	-0.7327839963
H	0.0000000000	0.0000000000	1.7915473038
F	0.0000000000	0.0000000000	2.7196083396

HGa···HCl

CCSD(T)-F12C/USERDEF ENERGY=-719.70887717

H	0.0000000000	0.0000000000	-2.9734303619
Ga	0.0000000000	0.0000000000	-1.3318431070
H	0.0000000000	0.0000000000	1.3782918217
Cl	0.0000000000	0.0000000000	2.6644827056

HGa···HBr

CCSD(T)-F12C/USERDEF ENERGY=-675.66158813

H	0.0000000000	0.0000000000	-3.8364031363
Ga	0.0000000000	0.0000000000	-2.1929166197
H	0.0000000000	0.0000000000	0.5249189095
Br	0.0000000000	0.0000000000	1.9551952861

HGa···HI

CCSD(T)-F12C/USERDEF ENERGY=-554.78510508

H	0.0000000000	0.0000000000	-4.5113184978
Ga	0.0000000000	0.0000000000	-2.8633651222
H	0.0000000000	0.0000000000	-0.0147224353
I	0.0000000000	0.0000000000	1.6090394421

HGa···HCCH

CCSD(T)-F12C/USERDEF ENERGY=-336.55705317

H	0.0000000000	0.0000000000	-2.9505597221
Ga	0.0000000000	0.0000000000	-1.2988465068
H	0.0000000000	0.0000000000	1.9234475344
C	0.0000000000	0.0000000000	2.9891953596
C	0.0000000000	0.0000000000	4.1951283174
H	0.0000000000	0.0000000000	5.2581842418

HGa···HCP

CCSD(T)-F12C/USERDEF ENERGY=-638.85309973

H	0.0000000000	0.0000000000	-3.6868569709
Ga	0.0000000000	0.0000000000	-2.0351476553
H	0.0000000000	0.0000000000	1.1731137458
C	0.0000000000	0.0000000000	2.2473452246
P	0.0000000000	0.0000000000	3.7913166946

HIn···HX complexes

HIn···HF

CCSD(T)-F12C/USERDEF ENERGY=-290.69398618

In	0.0000000000	0.0000000000	0.5120186618
H	0.0000000000	0.0000000000	2.3209739682
H	0.0000000000	0.0000000000	-2.1750435489
F	0.0000000000	0.0000000000	-3.1022118996

HIn···HCl

CCSD(T)-F12C/USERDEF ENERGY=-650.67486336

In	0.0000000000	0.0000000000	0.9728803539
H	0.0000000000	0.0000000000	2.7907999134
H	0.0000000000	0.0000000000	-1.8908165889
Cl	0.0000000000	0.0000000000	-3.1764096531

HIn···HBr

CCSD(T)-F12C/USERDEF ENERGY=-606.62768164

In	0.0000000000	0.0000000000	1.7501277218
H	0.0000000000	0.0000000000	3.5699943165
H	0.0000000000	0.0000000000	-1.1160036287
Br	0.0000000000	0.0000000000	-2.5458442680

HIn···HI

CCSD(T)-F12C/USERDEF ENERGY=-485.75135194

In	0.0000000000	0.0000000000	2.4048913897
H	0.0000000000	0.0000000000	4.2300674270
H	0.0000000000	0.0000000000	-0.5808937711
I	0.0000000000	0.0000000000	-2.2048532013

HIn···HCCH

CCSD(T)-F12C/USERDEF ENERGY=-267.52321560

In	0.0000000000	0.0000000000	0.9158144755
H	0.0000000000	0.0000000000	2.7451417408
H	0.0000000000	0.0000000000	-2.4764098179
C	0.0000000000	0.0000000000	-3.5419121349
C	0.0000000000	0.0000000000	-4.7477937474
H	0.0000000000	0.0000000000	-5.8108879254

HIn···HCP

CCSD(T)-F12C/USERDEF ENERGY=-569.81928300

In	0.0000000000	0.0000000000	1.5040538343
H	0.0000000000	0.0000000000	3.3333738903
H	0.0000000000	0.0000000000	-1.8655024704
C	0.0000000000	0.0000000000	-2.9395403424
P	0.0000000000	0.0000000000	-4.4834133319

Table S2. Optimised geometries (Å) and energies (au) of $H_2M\cdots HX$ complexes (M= C, Si, Ge, Sn) calculated at the CCSD(T)(F12c)/cc-pVDZ-F12 level.

$H_2C\cdots HX$ complexes

$H_2C\cdots HF$

CCSD(T)-F12C/CC-PVDZ-F12 ENERGY=-139.46023858

C	0.0000000000	0.0000000000	-1.5745032183
H	0.8717023601	0.0000000000	-2.2484469529
H	-0.8717023601	0.0000000000	-2.2484469529
H	0.0000000000	0.0000000000	0.2760666580
F	0.0000000000	0.0000000000	1.2193497933

$H_2C\cdots HCl$

CCSD(T)-F12C/CC-PVDZ-F12 ENERGY=-499.43703015

C	0.0000000000	0.0000000000	-2.3415639042
H	0.8694007458	0.0000000000	-3.0217853668
H	-0.8694007458	0.0000000000	-3.0217853668
H	0.0000000000	0.0000000000	-0.3288749752
Cl	0.0000000000	0.0000000000	0.9744610324

$H_2C\cdots HBr$

CCSD(T)-F12C/USERDEF ENERGY=-455.38882008

C	0.0000000000	0.0000000000	-2.9246511111
H	0.8699247648	0.0000000000	-3.6042829435
H	-0.8699247648	0.0000000000	-3.6042829435
H	0.0000000000	0.0000000000	-0.9073691806
Br	0.0000000000	0.0000000000	0.5420049070

$H_2C\cdots HI$

CCSD(T)-F12C/USERDEF ENERGY=-334.51062014

C	0.0000000000	0.0000000000	-3.4142569977
H	0.8674003480	0.0000000000	-4.0991512067
H	-0.8674003480	0.0000000000	-4.0991512067
H	0.0000000000	0.0000000000	-1.2380487505
I	0.0000000000	0.0000000000	0.3980911497

$H_2C\cdots HCCH$

CCSD(T)-F12C/CC-PVDZ-F12 ENERGY=-116.28206015

C	0.0000000000	0.0000000000	-2.6754987491
H	0.8627862382	0.0000000000	-3.3685654765
H	-0.8627862382	0.0000000000	-3.3685654765
H	0.0000000000	0.0000000000	-0.1778324419
C	0.0000000000	0.0000000000	0.8918359025
C	0.0000000000	0.0000000000	2.0986392389
H	0.0000000000	0.0000000000	3.1615837814

$H_2C\cdots HCP$

CCSD(T)-F12C/CC-PVDZ-F12 ENERGY=-418.57795766

C	0.0000000000	0.0000000000	-3.5004277339
H	0.8635861526	0.0000000000	-4.1927479942
H	-0.8635861526	0.0000000000	-4.1927479942
H	0.0000000000	0.0000000000	-0.9929816657
C	0.0000000000	0.0000000000	0.0845661344
P	0.0000000000	0.0000000000	1.6297942658

$H_2Si\cdots HX$ complexes

$H_2Si\cdots HF$

CCSD(T)-F12C/CC-PVDZ-F12 ENERGY=-390.55806045

Si	0.0000000000	0.0000000000	-1.3001110451
H	0.0000000000	1.1088808831	-2.3209471984
H	0.0000000000	-1.1088808831	-2.3209471984
H	0.0000000000	0.0000000000	1.1774756258
F	0.0000000000	0.0000000000	2.1057662064

$H_2Si\cdots HCl$

CCSD(T)-F12C/CC-PVDZ-F12 ENERGY=-750.53832086

Si	0.0000000000	0.0000000000	-2.1268385432
H	0.0000000000	1.1034290306	-3.1579057392
H	0.0000000000	-1.1034290306	-3.1579057392
H	0.0000000000	0.0000000000	0.5634696432
Cl	0.0000000000	0.0000000000	1.8484003987

$H_2Si\cdots HBr$

CCSD(T)-F12C/USERDEF ENERGY=-706.49091841

Si	0.0000000000	0.0000000000	-2.7841445909
H	0.0000000000	1.1024489915	-3.8174804477
H	0.0000000000	-1.1024489915	-3.8174804477
H	0.0000000000	0.0000000000	-0.0660507109
Br	0.0000000000	0.0000000000	1.3621771972

$H_2Si\cdots HI$

CCSD(T)-F12C/USERDEF ENERGY=-585.61439124

Si	0.0000000000	0.0000000000	-2.8615481730
H	0.0000000000	1.0996788333	-3.9000467852
H	0.0000000000	-1.0996788333	-3.9000467852
H	0.0000000000	0.0000000000	0.0086406206
I	0.0000000000	0.0000000000	1.6300221229

$H_2Si\cdots HCCH$

CCSD(T)-F12C/CC-PVDZ-F12 ENERGY=-367.38665621

Si	0.0000000000	0.0000000000	-2.2012927770
H	0.0000000000	1.0967287845	-3.2445344064
H	0.0000000000	-1.0967287845	-3.2445344064
H	0.0000000000	0.0000000000	0.9571038263
C	0.0000000000	0.0000000000	2.0227530262
C	0.0000000000	0.0000000000	3.2286445631
H	0.0000000000	0.0000000000	4.2916846545

$H_2Si\cdots HCP$

CCSD(T)-F12C/CC-PVDZ-F12 ENERGY=-669.68267626

Si	0.0000000000	0.0000000000	-3.1110964930
H	0.0000000000	1.0966626869	-4.1544250942
H	0.0000000000	-1.0966626869	-4.1544250942
H	0.0000000000	0.0000000000	0.0400928949
C	0.0000000000	0.0000000000	1.1141300827
P	0.0000000000	0.0000000000	2.6580336180

H_2Ge···HX complexes

H_2Ge···HF

CCSD(T)-F12C/USERDEF ENERGY=-395.02153561

Ge	0.0000000000	0.0000000000	-0.6944919388
H	0.0000000000	1.1520946453	-1.7767826277
H	0.0000000000	-1.1520946453	-1.7767826277
H	0.0000000000	0.0000000000	1.8183726442
F	0.0000000000	0.0000000000	2.7456065585

H_2Ge···HCl

CCSD(T)-F12C/USERDEF ENERGY=-755.00215298

Ge	0.0000000000	0.0000000000	-1.2830932983
H	0.0000000000	1.1475764407	-2.3767897805
H	0.0000000000	-1.1475764407	-2.3767897805
H	0.0000000000	0.0000000000	1.4374666209
Cl	0.0000000000	0.0000000000	2.7214110343

H_2Ge···HBr

CCSD(T)-F12C/USERDEF ENERGY=-710.95484024

Ge	0.0000000000	0.0000000000	-2.1498264762
H	0.0000000000	1.1468218017	-3.2458674112
H	0.0000000000	-1.1468218017	-3.2458674112
H	0.0000000000	0.0000000000	0.6004924587
Br	0.0000000000	0.0000000000	2.0273568624

H_2Ge···HI

CCSD(T)-F12C/USERDEF ENERGY=-590.07846112

Ge	0.0000000000	0.0000000000	-2.8369896928
H	0.0000000000	1.1444316614	-3.9382405416
H	0.0000000000	-1.1444316614	-3.9382405416
H	0.0000000000	0.0000000000	0.0645142495
I	0.0000000000	0.0000000000	1.6848067588

H_2Ge···HCCH

CCSD(T)-F12C/USERDEF ENERGY=-371.85077564

Ge	0.0000000000	0.0000000000	-1.2314993520
H	0.0000000000	1.1414898500	-2.3376473394
H	0.0000000000	-1.1414898500	-2.3376473394
H	0.0000000000	0.0000000000	1.9460356240
C	0.0000000000	0.0000000000	3.0113883927
C	0.0000000000	0.0000000000	4.2172521160
H	0.0000000000	0.0000000000	5.2803402847

H_2Ge···HCP

CCSD(T)-F12C/USERDEF ENERGY=-674.14678723

Ge	0.0000000000	0.0000000000	-1.9532196058
H	0.0000000000	1.1413765732	-3.0595392788
H	0.0000000000	-1.1413765732	-3.0595392788
H	0.0000000000	0.0000000000	1.2270433810
C	0.0000000000	0.0000000000	2.3007826628
P	0.0000000000	0.0000000000	3.8445570882

$H_2Sn\cdots HX$ complexes

$H_2Sn\cdots HF$

CCSD(T)-F12C/USERDEF ENERGY=-315.45988025

Sn	0.0000000000	0.0000000000	-0.7151084519
H	0.0000000000	1.2653966630	-1.9203463109
H	0.0000000000	-1.2653966630	-1.9203463109
H	0.0000000000	0.0000000000	1.9729481076
F	0.0000000000	0.0000000000	2.8987749747

$H_2Sn\cdots HCl$

CCSD(T)-F12C/USERDEF ENERGY=-675.44095016

Sn	0.0000000000	0.0000000000	-1.2993372885
H	0.0000000000	1.2614682671	-2.5155063461
H	0.0000000000	-1.2614682671	-2.5155063461
H	0.0000000000	0.0000000000	1.5847899037
Cl	0.0000000000	0.0000000000	2.8677648730

$H_2Sn\cdots HBr$

CCSD(T)-F12C/USERDEF ENERGY=-631.39379139

Sn	0.0000000000	0.0000000000	-1.2947431497
H	0.0000000000	1.2610636644	-2.5129095545
H	0.0000000000	-1.2610636644	-2.5129095545
H	0.0000000000	0.0000000000	1.6083329364
Br	0.0000000000	0.0000000000	3.0344341183

$H_2Sn\cdots HI$

CCSD(T)-F12C/USERDEF ENERGY=-510.51757429

Sn	0.0000000000	0.0000000000	-1.3447948057
H	0.0000000000	1.2591598412	-2.5679701596
H	0.0000000000	-1.2591598412	-2.5679701596
H	0.0000000000	0.0000000000	1.6913435674
I	0.0000000000	0.0000000000	3.3115963533

$H_2Sn\cdots HCCH$

CCSD(T)-F12C/USERDEF ENERGY=-292.28986664

Sn	0.0000000000	0.0000000000	-1.2781189367
H	0.0000000000	1.2569810804	-2.5058437743
H	0.0000000000	-1.2569810804	-2.5058437743
H	0.0000000000	0.0000000000	2.0420439053
C	0.0000000000	0.0000000000	3.1071093875
C	0.0000000000	0.0000000000	4.3129135989
H	0.0000000000	0.0000000000	5.3759619801

$H_2Sn\cdots HCP$

CCSD(T)-F12C/USERDEF ENERGY=-594.58585968

Sn	0.0000000000	0.0000000000	-1.9839883658
H	0.0000000000	1.2569716257	-3.2117380728
H	0.0000000000	-1.2569716257	-3.2117380728
H	0.0000000000	0.0000000000	1.3389184116
C	0.0000000000	0.0000000000	2.4124443497
P	0.0000000000	0.0000000000	3.9561867187

Table S3. Optimised geometries (Å) and energies (au) of $H_3A \cdots HX$ complexes (A= N, P, As, Sb) calculated at the CCSD(T)(F12c)/cc-pVDZ-F12 level.

$H_3N \cdots HX$ complexes

$H_3N \cdots HF$

CCSD(T)-F12C/CC-PVDZ-F12 ENERGY=-156.88584303 au

H	-0.0000000000	0.0000000000	0.3270183318
F	0.0000000000	0.0000000000	1.2756597006
N	-0.0000000000	0.0000000000	-1.3757765561
H	0.9403605202	0.0000000000	-1.7499699304
H	-0.4701802601	-0.8143760991	-1.7499699304
H	-0.4701802601	0.8143760991	-1.7499699304

$H_3N \cdots HCl$

CCSD(T)-F12C/CC-PVDZ-F12 ENERGY=-516.86200051 au

H	-0.0000000000	0.0000000000	-0.2700093401
Cl	0.0000000000	0.0000000000	1.0439695504
N	-0.0000000000	0.0000000000	-2.0904991086
H	0.9406491026	0.0000000000	-2.4645433634
H	-0.4703245513	-0.8146260189	-2.4645433634
H	-0.4703245513	0.8146260189	-2.4645433634

$H_3N \cdots HBr$

CCSD(T)-F12C/CC-PVDZ-F12 ENERGY=-472.81370121

H	-0.0000000000	0.0000000000	-0.8706849389
Br	-0.0000000000	0.0000000000	0.5942106111
N	-0.0000000000	0.0000000000	-2.6705557333
H	0.9419455354	0.0000000000	-3.0413734882
H	-0.4709727677	-0.8157487626	-3.0413734882
H	-0.4709727677	0.8157487626	-3.0413734882

$H_3N \cdots HI$

CCSD(T)-F12C/USERDEF ENERGY=-351.93494575

H	-0.0000000000	0.0000000000	-1.2112820550
I	-0.0000000000	0.0000000000	0.4394256185
N	0.0000000000	0.0000000000	-3.1365122187
H	0.4705595775	-0.8150330962	-3.5095999602
H	-0.9411191549	0.0000000000	-3.5095999602
H	0.4705595775	0.8150330962	-3.5095999602

$H_3N \cdots HCCH$

CCSD(T)-F12C/[CP]cc-pVDZ-F12 Energy: -133.705397242332

H	-0.0000000000	0.0000000000	2.4474568933
C	-0.0000000000	0.0000000000	1.3845159147
C	0.0000000000	0.0000000000	0.1773186890
H	0.0000000000	0.0000000000	-0.8945275620
N	-0.0000000000	0.0000000000	-3.1818944622
H	0.9368801460	0.0000000000	-3.5657054487
H	-0.4684400730	-0.8113620067	-3.5657054487
H	-0.4684400730	0.8113620067	-3.5657054487

$H_3N\cdots HCP$

CCSD(T)-F12C/CC-PVDZ-F12 ENERGY=-436.00153715

P	-0.0000000000	0.0000000000	1.8797427999
C	-0.0000000000	0.0000000000	0.3341631584
H	-0.0000000000	0.0000000000	-0.7453218788
N	0.0000000000	0.0000000000	-3.0387037342
H	0.9368521714	0.0000000000	-3.4226832089
H	-0.4684260858	-0.8113377800	-3.4226832089
H	-0.4684260858	0.8113377800	-3.4226832089

$H_3P\cdots HX$ complexes

$H_3P\cdots HF$

CCSD(T)-F12C/CC-PVDZ-F12 ENERGY=-443.08563832

H	-0.0000000000	0.0000000000	1.1970626828
F	-0.0000000000	0.0000000000	2.1253710927
P	0.0000000000	0.0000000000	-1.1587965320
H	1.2042563695	0.0000000000	-1.8899063725
H	-0.6021281848	-1.0429166086	-1.8899063725
H	-0.6021281848	1.0429166086	-1.8899063725

$H_3P\cdots HCl$

CCSD(T)-F12C/CC-PVDZ-F12 ENERGY=-803.06563829

H	-0.0000000000	0.0000000000	0.6253278788
Cl	0.0000000000	0.0000000000	1.9098489363
P	-0.0000000000	0.0000000000	-1.9437307411
H	1.1997267140	0.0000000000	-2.6861042600
H	-0.5998633570	-1.0389938119	-2.6861042600
H	-0.5998633570	1.0389938119	-2.6861042600

$H_3P\cdots HBr$

CCSD(T)-F12C/USERDEF ENERGY=-759.01809630

H	0.0000000000	0.0000000000	-0.1980265017
Br	0.0000000000	0.0000000000	1.2286725401
P	0.0000000000	0.0000000000	-2.8156413932
H	1.1988955113	0.0000000000	-3.5601494300
H	-0.5994477557	-1.0382739693	-3.5601494300
H	-0.5994477557	1.0382739693	-3.5601494300

$H_3P\cdots HI$

CCSD(T)-F12C/USERDEF ENERGY=- -638.14149098

H	-0.0000000000	0.0000000000	-0.6722001839
I	-0.0000000000	0.0000000000	0.9474668892
P	0.0000000000	0.0000000000	-3.4500235973
H	0.5981469851	-1.0360209686	-4.2002910931
H	-1.1962939702	0.0000000000	-4.2002910931
H	0.5981469851	1.0360209686	-4.2002910931

$H_3P\cdots HCCH$

CCSD(T)-F12C/CC-PVDZ-F12 ENERGY=-419.91392017

H	0.0000000000	0.0000000000	2.5377790787
C	0.0000000000	0.0000000000	1.4747702999
C	-0.0000000000	0.0000000000	0.2687743745
H	-0.0000000000	0.0000000000	-0.7970385541
P	-0.0000000000	0.0000000000	-3.7834209228
H	1.1938704749	0.0000000000	-4.5383467258
H	-0.5969352376	-1.0339221602	-4.5383467259
H	-0.5969352376	1.0339221602	-4.5383467259

$H_3P\cdots HCP$

CCSD(T)-F12C/CC-PVDZ-F12 ENERGY=-722.20992032

P	-0.0000000000	0.0000000000	1.8373420991
C	0.0000000000	0.0000000000	0.2933717167
H	0.0000000000	0.0000000000	-0.7807443650
P	-0.0000000000	0.0000000000	-3.7669185582
H	1.1938787632	0.0000000000	-4.5219286679
H	-0.5969393815	-1.0339293379	-4.5219286680
H	-0.5969393815	1.0339293379	-4.5219286680

$H_3As\cdots HX$ complexes

$H_3As\cdots HF$

CCSD(T)-F12C/USERDEF ENERGY=-433.52198541

As	-0.0000000000	0.0000000000	-0.6595682663
H	1.2787535818	0.0000000000	-1.4680446248
H	-0.6393767909	-1.1074330870	-1.4680446248
H	-0.6393767909	1.1074330870	-1.4680446248
H	-0.0000000000	0.0000000000	1.8126794425
F	0.0000000000	0.0000000000	2.7385428345

$H_3As\cdots HCl$

CCSD(T)-F12C/USERDEF ENERGY=-793.50271243

As	0.0000000000	0.0000000000	-1.2310460773
H	1.2741057217	0.0000000000	-2.0514631513
H	-0.6370528609	-1.1034079221	-2.0514631513
H	-0.6370528609	1.1034079221	-2.0514631513
H	-0.0000000000	0.0000000000	1.4529569282
Cl	-0.0000000000	0.0000000000	2.7351903559

$H_3As\cdots HBr$

CCSD(T)-F12C/USERDEF ENERGY=-749.45536743

As	-0.0000000000	0.0000000000	-2.0895014785
H	1.2730407313	0.0000000000	-2.9127312551
H	-0.6365203656	-1.1024856133	-2.9127312551
H	-0.6365203656	1.1024856133	-2.9127312551
H	-0.0000000000	0.0000000000	0.6369332655
Br	0.0000000000	0.0000000000	2.0614034151

$H_3As \cdots HI$

CCSD(T)-F12C/USERDEF ENERGY=-628.57905733

As	0.0000000000	0.0000000000	-2.7734489787
H	1.2707219254	0.0000000000	-3.6023974402
H	-0.6353609627	-1.1004774685	-3.6023974402
H	-0.6353609627	1.1004774685	-3.6023974402
H	0.0000000000	0.0000000000	0.1040163237
I	-0.0000000000	0.0000000000	1.7223805623

$H_3As \cdots HCCH$

CCSD(T)-F12C/USERDEF ENERGY=-410.35155110

As	-0.0000000000	0.0000000000	-1.1669338117
H	1.2685499748	0.0000000000	-2.0000191752
H	-0.6342749874	-1.0985965041	-2.0000191752
H	-0.6342749874	1.0985965041	-2.0000191752
H	0.0000000000	0.0000000000	1.9220475971
C	0.0000000000	0.0000000000	2.9871891880
C	0.0000000000	0.0000000000	4.1929933647
H	0.0000000000	0.0000000000	5.2560320594

$H_3As \cdots HCP$

CCSD(T)-F12C/USERDEF ENERGY=-712.64757007

As	0.0000000000	0.0000000000	-1.8628927710
H	1.2684988830	0.0000000000	-2.6962958097
H	-0.6342494415	-1.0985522573	-2.6962958097
H	-0.6342494415	1.0985522573	-2.6962958097
H	0.0000000000	0.0000000000	1.2220609639
C	-0.0000000000	0.0000000000	2.2956292276
P	-0.0000000000	0.0000000000	3.8393612349

$H_3Sb \cdots HX$ complexes

$H_3Sb \cdots HF$

CCSD(T)-F12C/USERDEF ENERGY=-341.54015717

Sb	-0.0000000000	0.0000000000	-0.4761368426
H	1.4348817651	0.0000000000	-1.3985852997
H	-0.7174408826	-1.2426440600	-1.3985852997
H	-0.7174408826	1.2426440600	-1.3985852997
H	-0.0000000000	0.0000000000	2.2309800524
F	-0.0000000000	0.0000000000	3.1555303231

$H_3Sb \cdots HCl$

CCSD(T)-F12C/USERDEF ENERGY=-701.52142563

Sb	-0.0000000000	0.0000000000	-0.9239326458
H	1.4301942955	0.0000000000	-1.8586850011
H	-0.7150971477	-1.2385845922	-1.8586850011
H	-0.7150971477	1.2385845922	-1.8586850011
H	-0.0000000000	0.0000000000	1.9936643491
Cl	-0.0000000000	0.0000000000	3.2747478198

$H_3Sb\cdots HBr$

CCSD(T)-F12C/USERDEF ENERGY=-657.47426433

Sb	-0.0000000000	0.0000000000	-1.7012209687
H	1.4292290244	0.0000000000	-2.6385787729
H	-0.7146145122	-1.2377486429	-2.6385787729
H	-0.7146145122	1.2377486429	-2.6385787729
H	-0.0000000000	0.0000000000	1.2525921566
Br	-0.0000000000	0.0000000000	2.6762077301

$H_3Sb\cdots HI$

CCSD(T)-F12C/USERDEF ENERGY=-536.59817558

Sb	-0.0000000000	0.0000000000	-2.3685067167
H	1.4270394716	0.0000000000	-3.3115482781
H	-0.7135197358	-1.2358524346	-3.3115482781
H	-0.7135197358	1.2358524346	-3.3115482781
H	-0.0000000000	0.0000000000	0.7274749589
I	-0.0000000000	0.0000000000	2.3454160939

$H_3Sb\cdots HCCH$

CCSD(T)-F12C/USERDEF ENERGY=-318.37068610

Sb	-0.0000000000	0.0000000000	-0.8444255729
H	1.4251832779	0.0000000000	-1.7912104339
H	-0.7125916389	-1.2342449237	-1.7912104339
H	-0.7125916389	1.2342449237	-1.7912104339
H	0.0000000000	0.0000000000	2.4889432600
C	0.0000000000	0.0000000000	3.5536663612
C	0.0000000000	0.0000000000	4.7593581671
H	0.0000000000	0.0000000000	5.8224381985

$H_3Sb\cdots HCP$

CCSD(T)-F12C/USERDEF ENERGY=-620.66670953

Sb	-0.0000000000	0.0000000000	-1.4073427638
H	1.4250761855	0.0000000000	-2.3543389094
H	-0.7125380927	-1.2341521789	-2.3543389094
H	-0.7125380927	1.2341521789	-2.3543389094
H	0.0000000000	0.0000000000	1.9213023823
C	0.0000000000	0.0000000000	2.9944670751
P	0.0000000000	0.0000000000	4.5380340329

Table S4. Optimised geometries (Å) and energies (au) of $H_2A\cdots HX$ complexes (A= O, S, Se, Te) calculated at the CCSD(T)(F12c)/cc-pVDZ-F12 level.

$H_2O\cdots HX$ complexes

$H_2O\cdots HF$

CCSD(T)-F12C/CC-PVDZ-F12 ENERGY=-176.74718924

H	0.0000000000	0.0407698523	0.3709229786
F	0.0000000000	-0.0067455140	1.3030327060
O	0.0000000000	0.0523280940	-1.3496858121
H	0.7631047850	-0.3676026810	-1.7511263922
H	-0.7631047850	-0.3676026810	-1.7511263922

$H_2O\cdots HCl$

CCSD(T)-F12C/CC-PVDZ-F12 ENERGY=-536.72486041

H	0.0000000000	0.0420146537	-0.1895332626
Cl	0.0000000000	-0.0033682429	1.0984813678
O	0.0000000000	0.0472534271	-2.1019859393
H	0.7615813554	-0.3370279670	-2.5401910667
H	-0.7615813554	-0.3370279670	-2.5401910667

$H_2O\cdots HBr$

CCSD(T)-F12C/USERDEF ENERGY=-492.67656764

H	0.0000000000	0.0491812446	-0.7869901303
Br	0.0000000000	-0.0014584408	0.6419812236
O	0.0000000000	0.0476640029	-2.7556804072
H	0.7611242932	-0.3448188446	-3.1875974243
H	-0.7611242932	-0.3448188446	-3.1875974243

$H_2O\cdots HI$

CCSD(T)-F12C/USERDEF ENERGY=-371.79904495

H	0.0000000000	0.0377654455	-1.1418751690
I	0.0000000000	-0.0008301315	0.4790165907
O	0.0000000000	0.0426759100	-3.2583459589
H	0.7600978885	-0.3084676549	-3.7257700158
H	-0.7600978885	-0.3084676549	-3.7257700158

$H_2O\cdots HCCH$

CCSD(T)-F12C/CC-PVDZ-F12 ENERGY=-153.57180993

H	0.0000000000	0.1149066814	2.3157225325
C	0.0000000000	0.0618318928	1.2541211749
C	0.0000000000	-0.0005009257	0.0491578856
H	0.0000000000	-0.0546333478	-1.0174357695
O	0.0000000000	-0.1726755003	-3.2131356768
H	0.7595370069	-0.2045792994	-3.7966396815
H	-0.7595370069	-0.2045792994	-3.7966396815

$H_2O\cdots HCP$

CCSD(T)-F12C/CC-PVDZ-F12 ENERGY=-455.86765039

P	0.0000000000	0.0551108175	1.7825446617
C	0.0000000000	-0.0135050596	0.2393446277
H	0.0000000000	-0.0625491918	-0.8352714994
O	0.0000000000	-0.1640534174	-3.0458385646

H	0.7593848408	-0.1946164735	-3.6297336553
H	-0.7593848408	-0.1946164735	-3.6297336553

$H_2S\cdots HX$ complexes

$H_2S\cdots HF$

CCSD(T)-F12C/CC-PVDZ-F12 ENERGY=-499.33223495

H	0.0000000000	0.0499940268	1.1165547949
F	0.0000000000	-0.0068387636	2.0430272851
S	0.0000000000	0.0565055049	-1.1673964877
H	0.9665407245	-0.8606818436	-1.2849704274
H	-0.9665407245	-0.8606818436	-1.2849704274

$H_2S\cdots HCl$

CCSD(T)-F12C/CC-PVDZ-F12 ENERGY=-859.31173209

H	0.0000000000	-0.0593418492	0.6260000154
Cl	0.0000000000	0.1186864122	1.9009802777
S	0.0000000000	0.0539396203	-1.9357398273
H	0.9815129425	-0.8597149545	-2.0841440496
H	-0.9815129425	-0.8597149545	-2.0841440496

$H_2S\cdots HBr$

CCSD(T)-F12C/[CP]USERDEF Energy: -815.264261205547

H	0.0000000000	0.0454134223	-0.2404282764
Br	0.0000000000	-0.0010738344	1.1861990014
S	0.0000000000	0.0553959963	-2.7667700747
H	0.9652490923	-0.8611454943	-2.8955389900

$H_2S\cdots HI$

CCSD(T)-F12C/[CP]USERDEF Energy: -694.38792909

H	0.0000000000	0.0440309020	-0.7062623213
I	0.0000000000	-0.0005443137	0.9137681769
S	0.0000000000	0.0551419496	-3.3761294380
H	0.9651474451	-0.8647117228	-3.4781515645
H	-0.9651474451	-0.8647117228	-3.4781515645

$H_2S\cdots HCCH$

CCSD(T)-F12C/CC-PVDZ-F12 ENERGY=-476.16038946

H	0.0000000000	0.0481666590	2.5185670059
C	0.0000000000	0.0838815588	1.4561384654
C	0.0000000000	0.1271443038	0.2507132673
H	0.0000000000	0.1630151804	-0.8148881495
S	0.0000000000	-0.0209027079	-3.6728184968
H	0.9649500811	-0.9420499508	-3.7622096281
H	-0.9649500811	-0.9420499508	-3.7622096281

$H_2S\cdots HCP$

CCSD(T)-F12C/CC-PVDZ-F12 ENERGY=-778.45634983

P	0.0000000000	0.0452430699	1.9531502342
C	0.0000000000	0.1034398719	0.4102514178
H	0.0000000000	0.1428735827	-0.6633866383
S	0.0000000000	0.0008847225	-3.5293709848

H	0.9648907882	-0.9176180775	-3.6401259166
H	-0.9648907882	-0.9176180775	-3.6401259166

$H_2Se\cdots HX$ complexes

$H_2Se\cdots HF$

CCSD(T)-F12C/USERDEF ENERGY=-473.62854072

Se	0.0000000000	-0.0005807850	-0.0022217815
H	1.0451737627	0.0001559198	1.0234956241
H	-1.0451737627	0.0001559198	1.0234956241
H	0.0000000000	-2.4119583557	-0.2163083597
F	0.0000000000	-3.3375926989	-0.2763911070

$H_2Se\cdots HCl$

CCSD(T)-F12C/USERDEF ENERGY=-833.60888222

Se	0.0000000000	0.0276052674	0.0040784347
H	1.0444819857	0.0222951963	1.0301855918
H	-1.0444819857	0.0222951963	1.0301855918
H	0.0000000000	-2.5758242306	-0.2276136279
Cl	0.0000000000	-3.8578714293	-0.3133159904

$H_2Se\cdots HBr$

CCSD(T)-F12C/USERDEF ENERGY=-789.56143410

Se	0.0000000000	0.0269000627	0.0030916377
H	1.0442735015	0.0212213900	1.0292170849
H	-1.0442735015	0.0212213900	1.0292170849
H	0.0000000000	-2.6111803070	-0.2034596031
Br	0.0000000000	-4.0375125356	-0.2628862044

$H_2Se\cdots HI$

CCSD(T)-F12C/USERDEF ENERGY=-668.68487436

Se	0.0000000000	0.0832453647	0.0153928529
H	1.0439891941	0.0696969362	1.0415690245
H	-1.0439891941	0.0696969362	1.0415690245
H	0.0000000000	-2.6917793137	-0.2171041468
I	0.0000000000	-4.3102099234	-0.3062467550

$H_2Se\cdots HCCH$

CCSD(T)-F12C/USERDEF ENERGY=-450.45711014

Se	0.0000000000	0.0773450904	0.0147407183
H	1.0437463822	0.0705970778	1.0411490668
H	-1.0437463822	0.0705970778	1.0411490668
H	0.0000000000	-2.9206637012	-0.3475648434
C	0.0000000000	-3.9817280517	-0.4496164128
C	0.0000000000	-5.1824429915	-0.5626542846
H	0.0000000000	-6.2406145016	-0.6640133112

$H_2Se\cdots HCP$

CCSD(T)-F12C/USERDEF ENERGY=-752.75312010

Se	0.0000000000	0.0657558195	0.0128549858
H	1.0437182050	0.0579036048	1.0393105410
H	-1.0437182050	0.0579036048	1.0393105410
H	0.0000000000	-2.9324569458	-0.3398521252
C	0.0000000000	-4.0020453931	-0.4412337565
P	0.0000000000	-5.5391706902	-0.5862401861

$H_2Te\cdots HX$ complexes

$H_2Te\cdots HF$

CCSD(T)-F12C/USERDEF ENERGY=-368.72221907

Te	0.0000000000	-0.0187629118	-0.4708933638
H	1.1779854098	1.1389886132	-0.6195095496
H	-1.1779854098	1.1389886132	-0.6195095496
H	0.0000000000	0.0109572820	2.1858446627
F	0.0000000000	0.0045813794	3.1124537077

$H_2Te\cdots HCl$

CCSD(T)-F12C/USERDEF ENERGY=-728.70296236

Te	0.0000000000	-0.0185298819	-0.8966170089
H	1.1771321214	1.1447928102	-1.0002684341
H	-1.1771321214	1.1447928102	-1.0002684341
H	0.0000000000	-0.0042613240	1.9444559938
Cl	0.0000000000	0.0017189845	3.2286366876

$H_2Te\cdots HBr$

CCSD(T)-F12C/USERDEF ENERGY=-684.65566378

Te	0.0000000000	-0.0180439020	-1.6411090307
H	1.1768511302	1.1504154392	-1.6443746938
H	-1.1768511302	1.1504154392	-1.6443746938
H	0.0000000000	-0.0550626405	1.2202938106
Br	0.0000000000	0.0004856110	2.6468060601

$H_2Te\cdots HI$

CCSD(T)-F12C/USERDEF ENERGY=-563.77927966

Te	0.0000000000	-0.0180350146	-2.2847749134
H	1.1766095217	1.1506863350	-2.2773655525
H	-1.1766095217	1.1506863350	-2.2773655525
H	0.0000000000	-0.0413633756	0.7068462633
I	0.0000000000	0.0001837124	2.3278419673

$H_2Te\cdots HCCH$

CCSD(T)-F12C/USERDEF ENERGY=-345.55142390

Te	0.0000000000	-0.0182360523	-0.8270020457
H	1.1767005856	1.1464996183	-0.9221630509
H	-1.1767005856	1.1464996183	-0.9221630509
H	0.0000000000	-0.0165122493	2.4557605527
C	0.0000000000	-0.0052796657	3.5213409289
C	0.0000000000	0.0065338574	4.7271830256
H	0.0000000000	0.0171576513	5.7901765758

$H_2Te\cdots HCP$

CCSD(T)-F12C/USERDEF ENERGY=-647.84745694

Te	0.0000000000	-0.0180348235	-1.3701567272
H	1.1766999307	1.1471155240	-1.4614364219
H	-1.1766999307	1.1471155240	-1.4614364219
H	0.0000000000	-0.0239889920	1.9046602018
C	0.0000000000	-0.0115297776	2.9787085003
P	0.0000000000	0.0048899412	4.5225709710

Table S5. Optimised geometries (Å) and energies (au) of HA···HX complexes (A= F, Cl, Br, I) calculated at the CCSD(T)(F12c)/cc-pVDZ-F12 level.

HF···HX complexes

HF···HF

CCSD(T)-F12C/CC-PVDZ-F12 ENERGY=-200.75424512

H	0.0000000000	0.8081852005	-1.6841030525
F	0.0000000000	-0.0459371668	-1.3415003410
H	0.0000000000	-0.1298984348	0.4924381840
F	0.0000000000	0.0099513861	1.4047228491

HF···HCl

CCSD(T)-F12C/CC-PVDZ-F12 ENERGY=-560.73431059

H	0.0000000000	0.7805345446	-2.5335389560
F	0.0000000000	-0.0431649439	-2.1256762150
H	0.0000000000	-0.1641080138	-0.0544779396
Cl	0.0000000000	0.0056058455	1.2126764767

HF···HBr

CCSD(T)-F12C/USERDEF ENERGY=-516.68660196

H	0.0000000000	0.7733135214	-3.2633024894
F	0.0000000000	-0.0421068252	-2.8396280223
H	0.0000000000	-0.1811606540	-0.6832157948
Br	0.0000000000	0.0025418987	0.7249480774

HF···HI

CCSD(T)-F12C/USERDEF ENERGY=-395.81016295

H	0.0000000000	0.7402690075	-3.8513726604
F	0.0000000000	-0.0397285116	-3.3668025773
H	0.0000000000	-0.2029924458	-1.0591494053
I	0.0000000000	0.0016802731	0.5430295621

HF···HCCH

CCSD(T)-F12C/CC-PVDZ-F12 ENERGY=-177.58339531

H	0.0000000000	0.6608176912	-2.8102029300
F	0.0000000000	-0.0381954836	-2.2147808870
H	0.0000000000	-0.0217234321	0.0569581846
C	0.0000000000	-0.0037750061	1.1219434679
C	0.0000000000	0.0088745736	2.3277907081
H	0.0000000000	0.0200742283	3.3907258066

HF···HCP

CCSD(T)-F12C/CC-PVDZ-F12 ENERGY=-479.87927356

H	0.0000000000	0.6206693040	-3.6759636825
F	0.0000000000	-0.0352081702	-3.0333614812
H	0.0000000000	-0.0166964917	-0.7413256142
C	0.0000000000	-0.0078416108	0.3318545127
P	0.0000000000	0.0049821603	1.8756354374

HCl···HX complexes

HCl···HF

CCSD(T)-F12C/CC-PVDZ-F12 ENERGY=-560.73480921 au

H -0.4289561604 0.0000000000 -0.0227074533
Cl 0.0812094718 0.0000000000 1.1477932803
H 2.2432491539 0.0000000000 0.3099238291
F 3.0314975347 0.0000000000 -0.1692096563

HCl···HCl

CCSD(T)-F12C/CC-PVDZ-F12 ENERGY=-920.71597458 au

H -0.3970394650 0.0000000000 -0.0514138184
Cl -0.0137710700 0.0000000000 1.1657730965
H 2.4212098028 0.0000000000 0.4087284046
Cl 3.5130612666 0.0000000000 -0.2572876827

HCl···HBr

CCSD(T)-F12C/USERDEF ENERGY=-876.66862459

H -0.4465025120 0.0000000000 -0.0392261695
Cl -0.0850643598 0.0000000000 1.1844346872
H 2.4113640545 0.0000000000 0.4134574127
Br 3.6436633516 0.0000000000 -0.2928659304

HCl···HI

CCSD(T)-F12C/USERDEF ENERGY=-755.79251683

H -0.5934101340 0.0000000000 -0.0100671725
Cl -0.2034723060 0.0000000000 1.2042786863
H 2.4487681863 0.0000000000 0.4178426288
I 3.8715747881 0.0000000000 -0.3462541425

HCl···HCCH

CCSD(T)-F12C/CC-PVDZ-F12 ENERGY=-537.56547199

H 0.0000000000 1.2304276863 -2.0199768575
Cl 0.0000000000 -0.0353713862 -1.8651329575
H 0.0000000000 -0.0201456992 0.9505086439
C 0.0000000000 -0.0041462266 2.0149753524
C 0.0000000000 0.0057667172 3.2206301628
H 0.0000000000 0.0145508841 4.2836485191

HCl···HCP

CCSD(T)-F12C/CC-PVDZ-F12 ENERGY=-839.86143274

H 0.0000000000 1.2287100489 -2.8829296863
Cl 0.0000000000 -0.0350137381 -2.7122700109
H 0.0000000000 -0.0217096158 0.1170238952
C 0.0000000000 -0.0074686669 1.1897758268
P 0.0000000000 0.0036955216 2.7331385767

HBr···HX complexes

HBr···HF

CCSD(T)-F12C/USERDEF ENERGY=-516.68743165

H	0.0000000000	1.3990680392	-0.7345250431
Br	0.0000000000	-0.0179128399	-0.6619045026
H	0.0000000000	-0.0331455340	1.8052521089
F	0.0000000000	0.0028707482	2.7270496757

HBr···HCl

CCSD(T)-F12C/USERDEF ENERGY=-876.66885630

H	0.0000000000	1.4009093588	-1.2392055032
Br	0.0000000000	-0.0174024269	-1.2173321167
H	0.0000000000	-0.0901856122	1.4617831205
Cl	0.0000000000	0.0019573131	2.7372961547

HBr···HBr

CCSD(T)-F12C/USERDEF ENERGY=-832.62159022

H	0.0000000000	1.4008647265	-2.0852217065
Br	0.0000000000	-0.0173151366	-2.0704635457
H	0.0000000000	-0.0973648677	0.6708188682
Br	0.0000000000	0.0008722846	2.0883053709

HBr···HI

CCSD(T)-F12C/USERDEF ENERGY=-711.74555171

H	0.0000000000	1.4008545071	-2.7582905403
Br	0.0000000000	-0.0171572651	-2.7479153953
H	0.0000000000	-0.1227359081	0.1399710464
I	0.0000000000	0.0006514085	1.7509778205

HBr···HCCH

CCSD(T)-F12C/USERDEF ENERGY=-493.51832769

H	0.0000000000	1.3952115550	-1.2518094510
Br	0.0000000000	-0.0177714103	-1.1346866724
H	0.0000000000	-0.0108756821	1.8632180482
C	0.0000000000	-0.0038050717	2.9277917211
C	0.0000000000	0.0048838226	4.1334315218
H	0.0000000000	0.0116300445	5.1961310398

HBr···HCP

CCSD(T)-F12C/USERDEF ENERGY=-795.81434407

H	0.0000000000	1.3956278628	-1.9358147785
Br	0.0000000000	-0.0164269124	-1.8080465927
H	0.0000000000	-0.0570724467	1.1938850724
C	0.0000000000	-0.0301120505	2.2665122637
P	0.0000000000	0.0104949577	3.8095115599

HI···HX complexes

HI···HF

CCSD(T)-F12C/USERDEF ENERGY=-395.81154030

H 0.0000000000 1.6002165867 -0.5445643096
I 0.0000000000 -0.0128711274 -0.4827844129
H 0.0000000000 -0.0274149066 2.2142537009
F 0.0000000000 0.0025331523 3.1363163686

HI···HCl

CCSD(T)-F12C/USERDEF ENERGY=-755.79320194

H 0.0000000000 1.6000672046 -0.9742789485
I 0.0000000000 -0.0127929033 -0.9173477691
H 0.0000000000 -0.0365483630 1.9766092925
Cl 0.0000000000 0.0013413626 3.2551856468

HI···HBr

CCSD(T)-F12C/USERDEF ENERGY=-711.74604858

H 0.0000000000 1.6007839888 -1.7228282156
I 0.0000000000 -0.0127392860 -1.6841830742
H 0.0000000000 -0.0338192151 1.2599561885
Br 0.0000000000 0.0004665312 2.6806977616

HI···HI

CCSD(T)-F12C/USERDEF ENERGY=-590.87009130

H 0.0000000000 1.6003161543 -2.3962293812
I 0.0000000000 -0.0127341613 -2.3442621413
H 0.0000000000 -0.0427813177 0.7417896995
I 0.0000000000 0.0003635162 2.3574024485

HI···HCCH

CCSD(T)-F12C/USERDEF ENERGY=-372.64276742

H 0.0000000000 1.5952735464 -0.9664996858
I 0.0000000000 -0.0129336046 -0.8320692573
H 0.0000000000 -0.0045622345 2.4256072866
C 0.0000000000 -0.0009381887 3.4900606896
C 0.0000000000 0.0035162412 4.6956848109
H 0.0000000000 0.0069822830 5.7586759230

HI···HCP

CCSD(T)-F12C/USERDEF ENERGY=-674.93879808

H 0.0000000000 1.5941573452 -1.5334081045
I 0.0000000000 -0.0121205847 -1.3780995525
H 0.0000000000 -0.0532807222 1.8761291408
C 0.0000000000 -0.0276286374 2.9487453524
P 0.0000000000 0.0102313709 4.4917198390

Table S6. Optimised geometries (Å) and energies (au) of Rg···HX complexes (Rg = Ne, Ar, Kr, Xe) calculated at the CCSD(T)(F12c)/cc-pVDZ-F12 level.

Ne···HX complexes

Ne···HF
CCSD(T)-F12C/CC-PVDZ-F12 ENERGY=-229.21823020
F 0.0000000000 0.0000000000 -0.7263673410
H 0.0000000000 0.0000000000 0.1910760794
Ne 0.0000000000 0.0000000000 2.5467274047

Ne···HCl
CCSD(T)-F12C/CC-PVDZ-F12 ENERGY=-589.20104263
Cl 0.0000000000 0.0000000000 -1.1651341186
H 0.0000000000 0.0000000000 0.1092165321
Ne 0.0000000000 0.0000000000 2.7137210706

Ne···HBr
CCSD(T)-F12C/USERDEF ENERGY=-545.15409518
Br 0.0000000000 0.0000000000 -1.3477345962
H 0.0000000000 0.0000000000 0.0693425690
Ne 0.0000000000 0.0000000000 2.7811955172

Ne···HI
CCSD(T)-F12C/USERDEF ENERGY=-424.27861874
I 0.0000000000 0.0000000000 -1.5187108366
H 0.0000000000 0.0000000000 0.0945244080
Ne 0.0000000000 0.0000000000 2.9242438107

Ne···HCCH
CCSD(T)-F12C/CC-PVDZ-F12 ENERGY=-206.05181456
H 0.0000000000 0.0000000000 -2.4851977097
C 0.0000000000 0.0000000000 -1.4221055146
C 0.0000000000 0.0000000000 -0.2168048699
H 0.0000000000 0.0000000000 0.8461398890
Ne 0.0000000000 0.0000000000 3.5528959759

Ne···HCP
CCSD(T)-F12C/CC-PVDZ-F12 ENERGY=-508.34779845
P 0.0000000000 0.0000000000 -1.7718158719
C 0.0000000000 0.0000000000 -0.2288207430
H 0.0000000000 0.0000000000 0.8428574344
Ne 0.0000000000 0.0000000000 3.5896910793

Ar···HX complexes

Ar···HF

CCSD(T)-F12C/CC-PVDZ-F12 ENERGY=-627.44230967

F	0.0000000000	0.0000000000	-0.5969040941
H	0.0000000000	0.0000000000	0.3212875648
Ar	0.0000000000	0.0000000000	2.8679147963

Ar···HCl

CCSD(T)-F12C/CC-PVDZ-F12 ENERGY=-987.42498003

Cl	0.0000000000	0.0000000000	-1.0596790066
H	0.0000000000	0.0000000000	0.2151403798
Ar	0.0000000000	0.0000000000	3.0795079898

Ar···HBr

CCSD(T)-F12C/USERDEF ENERGY=-943.37802385

Br	0.0000000000	0.0000000000	-1.2029678844
H	0.0000000000	0.0000000000	0.2144040275
Ar	0.0000000000	0.0000000000	3.0808621239

Ar···HI

CCSD(T)-F12C/USERDEF ENERGY=-822.50250755

I	0.0000000000	0.0000000000	-1.4840574017
H	0.0000000000	0.0000000000	0.1292325608
Ar	0.0000000000	0.0000000000	3.1271231079

Ar···HCCH

CCSD(T)-F12C/CC-PVDZ-F12 ENERGY=-604.27562490

H	0.0000000000	0.0000000000	-2.5680504728
C	0.0000000000	0.0000000000	-1.5049852358
C	0.0000000000	0.0000000000	-0.2996280696
H	0.0000000000	0.0000000000	0.7635535842
Ar	0.0000000000	0.0000000000	3.7370568965

Ar···HCP

CCSD(T)-F12C/CC-PVDZ-F12 ENERGY=-906.57163277

P	0.0000000000	0.0000000000	-1.8972082867
C	0.0000000000	0.0000000000	-0.3541966668
H	0.0000000000	0.0000000000	0.7176032633
Ar	0.0000000000	0.0000000000	3.6968587611

Kr···HX complexes

Kr···HF

CCSD(T)-F12C/USERDEF ENERGY=-562.78677704

Kr	0.0000000000	0.0000000000	-0.6742643699
H	0.0000000000	0.0000000000	1.9518819803
F	0.0000000000	0.0000000000	2.8705557135

Kr···HCl

CCSD(T)-F12C/USERDEF ENERGY=-922.76947772

Kr	0.0000000000	0.0000000000	-1.2463882909
H	0.0000000000	0.0000000000	1.6247601281
Cl	0.0000000000	0.0000000000	2.8998865557

Kr···HBr

CCSD(T)-F12C/USERDEF ENERGY=-878.72252530

Kr	0.0000000000	0.0000000000	0.0341454760
H	0.0000000000	0.0000000000	2.9841222844
Br	0.0000000000	0.0000000000	4.4017322395

Kr···HI

CCSD(T)-F12C/USERDEF ENERGY=-757.84700821

Kr	0.0000000000	0.0000000000	-0.0651961434
H	0.0000000000	0.0000000000	3.0158846276
I	0.0000000000	0.0000000000	4.6293115158

Kr···HCCH

CCSD(T)-F12C/USERDEF ENERGY=-539.62029179

Kr	0.0000000000	0.0000000000	-0.0589567428
H	0.0000000000	0.0000000000	3.0088855141
C	0.0000000000	0.0000000000	4.0720730454
C	0.0000000000	0.0000000000	5.2774599211
H	0.0000000000	0.0000000000	6.3405382621

Kr···HCP

CCSD(T)-F12C/USERDEF ENERGY=-841.91632218

Kr	0.0000000000	0.0000000000	-0.0389405034
H	0.0000000000	0.0000000000	3.0207443202
C	0.0000000000	0.0000000000	4.0925516344
P	0.0000000000	0.0000000000	5.6356445488

Xe···HX complexes

Xe···HF

CCSD(T)-F12C/USERDEF ENERGY=-428.85849365

Xe	0.0000000000	0.0000000000	0.1354513578
H	0.0000000000	0.0000000000	2.9726979871
F	0.0000000000	0.0000000000	3.8918506551

Xe···HCl

CCSD(T)-F12C/USERDEF ENERGY=-788.84117574

Xe	0.0000000000	0.0000000000	-0.0225402731
H	0.0000000000	0.0000000000	3.0135826001
Cl	0.0000000000	0.0000000000	4.2889576731

Xe···HBr

CCSD(T)-F12C/USERDEF ENERGY=-744.79425702

Xe	0.0000000000	0.0000000000	-0.0651514654
H	0.0000000000	0.0000000000	3.0336503019
Br	0.0000000000	0.0000000000	4.4515011634

Xe···HI

CCSD(T)-F12C/USERDEF ENERGY=-623.91873997

Xe	0.0000000000	0.0000000000	-0.1535960233
H	0.0000000000	0.0000000000	3.0598315812
I	0.0000000000	0.0000000000	4.6737644421

Xe···HCCH

CCSD(T)-F12C/USERDEF ENERGY=-405.69175983

Xe	0.0000000000	0.0000000000	-0.2446353350
H	0.0000000000	0.0000000000	3.0551669506
C	0.0000000000	0.0000000000	4.1185360615
C	0.0000000000	0.0000000000	5.3239361189
H	0.0000000000	0.0000000000	6.3869962041

Xe···HCP

CCSD(T)-F12C/USERDEF ENERGY=-707.98779944

Xe	0.0000000000	0.0000000000	-0.2095191141
H	0.0000000000	0.0000000000	3.0774711703
C	0.0000000000	0.0000000000	4.1494649016
P	0.0000000000	0.0000000000	5.6925830422

Table S7. De (kJ mol $^{-1}$) values calculated at CCSD(T)-F12c/cc-VDZ-F12 level.

HX	HB···HX	HAl···HX	HGa···HX	HIn···HX
HF	33.24	16.11	17.96	16.38
HCl	20.52	10.58	11.59	10.89
HBr	18.00	9.85	10.66	9.22
HI	12.59	7.50	8.00	6.79
HCCH	9.01	4.51	5.37	4.66
HCP	8.94	4.71	5.15	4.85
	H2C···HX	H2Si···HX	H2Ge···HX	H2Sn···HX
HF	42.73	18.29	15.91	14.46
HCl	26.37	11.35	9.94	9.60
HBr	22.93	10.08	8.93	9.03
HI	15.78	7.31	6.50	7.02
HCCH	11.74	5.04	4.45	4.61
HCP	11.48	5.13	4.56	4.62
	H3N···HX	H3P···HX	H3As···HX	H3Sb···HX
HF	50.96	19.34	14.86	11.44
HCl	32.72	11.87	9.28	7.30
HBr	29.06	10.35	8.79	6.51
HI	20.45	7.48	6.44	4.74
HCCH	14.72	5.62	4.45	3.42
HCP	14.28	5.67	4.56	3.59
	H2O···HX	H2S···HX	H2Se···HX	H2Te···HX
HF	35.43	20.33	18.78	16.28
HCl	21.34	12.72	12.18	10.82
HBr	17.84	11.12	11.28	9.94
HI	12.52	8.04	8.27	7.49
HCCH	11.64	6.18	5.81	5.03
HCP	11.24	6.13	5.87	5.18
	HF···HX	HCl···HX	HBr···HX	HI···HX
HF	18.70	12.00	10.75	9.46
HCl	11.27	7.81	7.28	6.66
HBr	8.99	6.48	6.18	5.67
HI	6.44	4.89	4.50	4.61
HCCH	6.60	4.20	3.73	3.31
HCP	6.32	4.18	3.82	3.46

HX	Ne···HX	Ar···HX	Kr···HX	Xe···HX
HF	0.83	2.01	2.32	2.66
HCl	0.57	1.53	1.88	2.18
HBr	0.52	1.45	1.72	2.06
HI	0.44	1.28	1.50	1.84
HCCH	0.44	1.04	1.20	1.33
HCP	0.45	1.12	1.33	1.57
